# Inflammation and Antiviral Immune Response Associated With Severe Progression of COVID-19

**DOI:** 10.3389/fimmu.2021.631226

**Published:** 2021-02-18

**Authors:** Qiong Zhang, Yuting Meng, Kaihang Wang, Xujun Zhang, Wenbiao Chen, Jifang Sheng, Yunqing Qiu, Hongyan Diao, Lanjuan Li

**Affiliations:** State Key Laboratory for Diagnosis and Treatment of Infectious Diseases, National Clinical Research Center for Infectious Diseases, Collaborative Innovation Center for Diagnosis and Treatment of Infectious Diseases, The First Affiliated Hospital, College of Medicine, Zhejiang University, Hangzhou, China

**Keywords:** COVID-19, SARS-CoV-2, transcriptome, inflammation, immune response, myeloid cells, T cells, interferons

## Abstract

Coronavirus disease-2019 (COVID-19) is a novel respiratory disease induced by severe acute respiratory syndrome coronavirus 2 (SARS-CoV-2). It remains poorly understood how the host immune system responds to the infection during disease progression. We applied microarray analysis of the whole genome transcriptome to peripheral blood mononuclear cells (PBMCs) taken from severe and mild COVID-19 patients as well as healthy controls. Functional enrichment analysis of genes associated with COVID-19 severity indicated that disease progression is featured by overactivation of myeloid cells and deficient T cell function. The upregulation of TLR6 and MMP9, which promote the neutrophils-mediated inflammatory response, and the downregulation of SKAP1 and LAG3, which regulate T cells function, were associated with disease severity. Importantly, the regulation of these four genes was absent in patients with influenza A (H1N1). And compared with stimulation with hemagglutinin (HA) of H1N1 virus, the regulation pattern of these genes was unique in PBMCs response to Spike protein of SARS-CoV-2 *ex vivo*. Our data also suggested that severe SARS-CoV-2 infection largely silenced the response of type I interferons (IFNs) and altered the proportion of immune cells, providing a potential mechanism for the hypercytokinemia. This study indicates that SARS-CoV-2 infection impairs inflammatory and immune signatures in patients, especially those at severe stage. The potential mechanisms underpinning severe COVID-19 progression include overactive myeloid cells, impaired function of T cells, and inadequate induction of type I IFNs signaling.

## Introduction

The current pandemic of coronavirus disease-2019 (COVID-19) caused by severe acute respiratory syndrome-related coronavirus 2 (SARS-CoV-2) has infected over 20 million patients with more than 800000 deaths worldwide as of August, 2020. Most patients demonstrated mild to moderate symptoms, such as fever, cough, and shortness of breath. However, about 20% of patients develop rapidly into severe or critical illness with acute respiratory distress syndrome (ARDS), severe hypoxemia, acute lung injury (ALI), systemic inflammatory response syndrome (SIRS), and multiple organ dysfunction syndrome (MODS) which can eventually result in fatality (1–3). Identifying risk factors for severe disease progression is critical for early detection and effective therapy of severe patients, but progress is still undermined by the limited understanding of SARS-CoV-2 pathogenesis and the lack of prognostic biomarkers for severe cases.

The robust inflammatory response called “hypercytokinemia” or “cytokine storm” is featured by the overproduction and release of more than 150 inflammatory cytokines and chemical mediators by immune and non-immune defense cells, which ultimately leads to the tissue injure (4). However, it is still unclear what initiates and dominates the disproportionate host immune response. Myeloid cells, which include monocytes, macrophages, and dendritic cells and granulocytes (which are further subdivided into neutrophils, eosinophils, and basophils) are crucial for mounting successful immune responses against viruses (5, 6). However, a wealth of previous findings have pointed out myeloid cells are also critical for the initiation, propagation, and amplification of hypercytokinemia (7). As the first line of defense against infection, type I interferons (IFNs) have been implicated in the regulation of myeloid cell activation and migration (8). The prompt and proper activation of type I IFNs not only effectively restricts virus replication and reduces immunopathological damage, but also limits the overactive inflammatory response. Elevated acute phase reactants and high levels of circulating proinflammatory cytokines in COVID-19 patients suggest that hypercytokinemia may be a hallmark of COVID-19 (9–11). Thus, detailing the immune profile of COVID-19 patients at different stages of disease progression is critical for understanding the pathogenesis of SARS-CoV-2 and developing effective treatments.

In this study, in order to identify the unique immune characteristics of severe SARS-CoV-2 infection, we compared the global gene expression profiles of COVID-19 patients in mild and severe stages with healthy subjects. We also identified and validated key genes specifically regulated by SARS-CoV-2 both *in vivo* and *ex vivo*, whose expression correlates with disease progression. These genes provide potent diagnosis markers for identifying patients at risk for severe infection.

## Materials and Methods

The study was approved by the Clinical Research Ethics Committee of The First Affiliated Hospital, School of Medicine, Zhejiang University (Approval notice 2020-38) for emerging infectious diseases. The peripheral blood mononuclear cells (PBMCs) were all acquired from existing samples collected during standard diagnostic tests.

### SARS-CoV-2 Patients, H1N1 Patients, and Healthy Subjects

PBMC samples from 53 confirmed COVID-19 patients and 24 healthy donors were collected from The First Affiliated Hospital, Zhejiang University School of Medicine from January 28 to February 20, 2020. All patients were determined by laboratory tests for COVID-19 by RT-PCR. COVID-19 patients were divided into severe and mild groups according to the diagnosis and treatment scheme for SARS-CoV-2. The “severe” diagnosis includes dyspnoea (respiratory rate (RR) ≥30 times/min), resting finger oxygen saturation ≤93%, and artery PaO2/FiO2 ≤300 mmHg (1 mmHg=0.133 kPa). Among the severe cases, 18 were male and 9 were female, with a median age of 50. Mild group included 17 males and 9 females, with a median age of 50. Healthy control (HC) groups contained 17 males and nine females, with a median age of 47.

A total number of 22 H1N1 patients with a median age of 26 and 19 HCs with a median age of 26 accordingly were collected from Tongde Hospital of Zhejiang Province from February 2 to March 30, 2019. All H1N1 patients were outpatients and identified for laboratory-confirmed H1N1 influenza.

Healthy donors were recruited from patients who visited hospitals for routine physical examination. Subjects with active cancer, primary immunodeficiency disorders, HIV infection or any respiratory symptoms were excluded. Subjects with a recent medical record of taking immunosuppressive medications, antibiotics or chemotherapeutic drugs were also excluded from the study. The detailed information of the patients and healthy subjects could be found in the demographic and clinical table (**Table 1** and **Table S1**).

**Table 1:** Demographic and clinical characteristics of study population.

	Severe (n = 27)	Mild (n = 26)	Healthy people (n = 24)	P-Value
**Age, years**	51.0 (19.0–74.0)	50.0 (13.0–70.0)	48.0 (25.0–76.0)	0.7550^a^
**Men**	18 (67.0%)	17 (65.0%)	14 (58.0%)	0.8053^a^
**Women**	9 (33.0%)	9 (35.0%)	10 (42.0%)	
**Any comorbidity**	15 (55.6%)	11 (42.3%)	0 (0%)	0.3348^a^
**Diabetes**	3 (11.1%)	4 (14.8%)	0 (0%)	0.6460^a^
**Hypertension**	13 (48.1%)	5 (19.2%)	0 (0%)	0.0263^a^
**Cardiovascular disease**	1 (3.7%)	1 (3.8%)	0 (0%)	0.9783^a^
**Chronic liver disease**	2 (7.4%)	3 (11.5%)	0 (0%)	0.6070^a^
**White blood cell count, 10^9^/L**	6.20 (1.6–20.8)	5.15 (2.1–17.0)	NA	0.4933
**Monocyte count, 10^9^/L**	0.30 (0.06–1.12)	0.36 (0.06–0.92)	NA	0.2128
**Lymphocyte count, 10^9^/L**	0.63 (0.20–1.2)	0.9 (0.2–2.0)	NA	0.1227
**Neutrophil count, 10^9^/L**	5.7 (0.9–19.2)	3.4 (1.4–19.0)	NA	0.3189
**Eosinophil count, 10^9^/L**	0.0 (0.0–0.08)	0.0 (0.0–0.17)	NA	0.009
**Basophil count, 10^9^/L**	0.01 (0.0–0.04)	0.0 (0.0–0.06)	NA	0.4866
**Monocyte (%)**	3.9 (1.4–13.6)	7.3 (2.1–21.4)	NA	0.0222
**Lymphocyte (%)**	9.9 (1.9–40.2)	15.5 (1.4–39.4)	NA	0.147
**Neutrophil (%)**	86.7 (55.8–94.8)	76.9 (40.6–94.4)	NA	0.0493
**Eosinophil (%)**	0.0 (0.0–0.5)	0.0 (0.0–4.4)	NA	0.0091
**Basophil (%)**	0.1 (0.0–0.4)	0.05 (0.0–0.6)	NA	0.7191

Data are median (IQR), n (%) or n/N (%), where N is the total number of patients in one group. p-values of ages and genders compared differences among severe, mild and healthy groups, others indicated differences between severe and mild groups. P-values were calculated using Mann-Whitney U test. ^a^P-values were calculated by chi-square test.

### PBMCs Collection and RNA Extraction

Whole blood samples from patients and healthy donors were drawn into vacutainer tubes. The Ficoll density gradient centrifugation method was used to separate the PBMCs. The collected PBMCs were then lysed in RNAiso Plus (TAKARA) and total RNA was extracted according to the manufacturer’s instructions.

### Microarray Analysis

The PBMCs of five patients from severe and mild group respectively along with 5 healthy controls (HCs) with matched distribution of ages and genders were picked and subjected to microarray analysis (**Table S1**). Microarray experiment was conducted using 15 RNA samples derived from groups of mild patients (n = 5), severe patients (n = 5), and healthy control (n = 5). Briefly, mRNA was purified from total RNA after removal of rRNA (mRNA-ONLY™ Eukaryotic mRNA Isolation Kit, Epicentre). Then, each sample was amplified and transcribed into fluorescent cRNA along the entire length of the transcripts without 3’ bias utilizing a random priming method (Arraystar Flash RNA Labeling Kit, Arraystar). The labeled cRNAs were purified by RNeasy Mini Kit (Qiagen). The concentration and specific activity of the labeled cRNAs (pmol Cy3/μg cRNA) were measured by NanoDrop ND-1000. Arraystar Human LncRNA Microarray V5.0 containing 21,174 coding transcripts was used for hybridizations. The hybridized arrays were washed, fixed and scanned with using the Agilent DNA Microarray Scanner (part number G2505C).

### Treatment of PBMCs With Spike-ECD and HA Protein

PBMCs collected from healthy subjects were incubated in Roswell Park Memorial Institute (RPMI) 1640 medium with 10% fetal bovine serum (Gibco), 100 units/ml penicillin, and 100 mg/ml streptomycin at 37°C in 5% CO2 (v/v). Indicated concentration of extra cellular domain truncation of SARS-CoV-2 Spike protein (GenScript) or Hemagglutinin/HA protein of Influenza A H1N1 (A/California/04/2009) (Sino Biological) was then added to the cultured PBMCs for 12 h, followed by RNA extraction and qRT-PCR assay.

### Stimulation and Co-Cultures of THP-1 With HuT 78

The human monocytic cell line THP-1 and the human T cell line HuT 78 were obtained from American Tissue Culture Collection. THP-1 cell line was maintained in RPMI-1640 supplemented with 10% fetal bovine serum. HuT 78 cells were cultured in Iscove’s Modified Dulbecco’s Medium (Gibco), supplemented with 20% fetal calf serum (Gibco). THP-1 was treated with 100 ng/ml phorbol 12-myristate 13-acetate (PMA), which was followed by stimulation with or without 100 ng/ml Spike-ECD for 24 h. Cells were then co-cultured with Hu78 cell line for another 24 h. Both cells were harvested and underwent RNA extraction and qRT-PCR assay.

### Quantitative RT-PCR Assay

cDNA was synthesized from 1 μg of total RNA isolated from PBMCs using HiScript II Q Select RT SuperMix (Vazyme). Quantitative real-time PCR was performed using SYBR qPCR Master Mix (Vazyme) and the amplifications were performed in QuantStudio 5 (Applied Biosystems). For quantification of gene expression, the 2^-ΔΔCt^ method was used. Glyceraldehyde-3-phosphate dehydrogenase (GAPDH) expression was used for normalization. Primers specific for human glyceraldehyde-3-phosphate dehydrogenase (GAPDH) mRNA were used to normalize samples. The 5’-3’ sequences of primer pairs were as follows: GAPDH mRNA primers, GTC TCC TCT GAC TTC AAC AGC G (forward) and ACC ACC CTG TTG CTG TAG CCA A (reverse); TLR6 mRNA primers, ACT GAC CTT CCT GGA TGT GGC A (forward) and TGA CCT CAT CTT CTG GCA GCT C (reverse); SKAP1 mRNA primers, CAG CCA GAT GAA CTG TCC TTC C (forward) and GGA ACA ATC CCA ACG AGG CTG T (reverse); MMP9 mRNA primers, GCC ACT ACT GTG CCT TTG AGT C (forward) and CCC TCA GAG AAT CGC CAG TAC T (reverse); LAG3 mRNA primers, GCA GTG TAC TTC ACA GAG CTG TC (forward) and AAG CCA AAG GCT CCA GTC ACC A (reverse); IRF7 mRNA primers, CCA CGC TAT ACC ATC TAC CTG G (forward) and GCT GCT ATC CAG GGA AGA CAC A (reverse); MAVS mRNA primers, ATG GTG CTC ACC AAG GTG TCT G (forward) and TCT CAG AGC TGC TGT CTA GCC A (reverse).

### Data Analysis

Agilent Feature Extraction software (version 11.0.1.1) was used to analyze acquired array images. Quantile normalization and subsequent data processing were performed with using the GeneSpring GX v12.1 software package (Agilent Technologies). After quantile normalization of the raw data, mRNAs that at least five out of 15 samples have flags in Present or Marginal (“All Targets Value”) were chosen for further data analysis. P values were adjusted with Benjamini-Hochberg false discovery rate (FDR) correction. Differentially expressed probes were filtered by an FDR ≤ 0.05 and mean fold- difference ≥ 2 and ≤ 2 between pairwise combinations of two groups.

For principal components analysis (PCA) and clustered heatmap, gene-level expression data were normalized under StandardScaler from sklearn. To identify differentially expressed pathways, gene set enrichment analysis (GSEA) was performed on using GSEA_4.0.3 software. The “hallmark” gene sets from the Molecular Signatures Database (MSigDB) version 7.1 were employed. GO analysis were applied to determine the roles of these differentially expressed mRNAs played in these biological pathways or GO terms. Unless otherwise stated, a p-value ≤ 0.01 was used as a threshold to determine whether a particular term was statistically enriched. Biological process categories derived from GO annotation (http://www.geneontology.org) were reclassified based on analysis of gene function using GeneCards (http://www.genecards.org) and PubMed (https://www.ncbi.nlm.nih.gov/pubmed). For protein-protein interaction (PPI) network construction, we used Search Tool for the Retrieval of Interacting Genes (STRING) database to recognize potential interactions between selected genes and other DEGs with a score > 900. Then the PPI network was visualized by Cytoscape software. GO analysis was conducted in the related DEGs. The nodes representing genes included in the most significantly enriched GO Term were drawn red, otherwise were drawn blue. The CIBERSORT (https://cibersortx.stanford.edu/) with the original CIBERSORT gene signatures file LM22 was employed for estimation abundances of member cell types by gene expression profile.

### Statistical Analysis

Continuous variables compared using the Mann-Whitney U test or the one-way ANOVA for multiple comparison. Categorical variables were expressed as number (%) and compared by chi-square test. Error bars represent standard deviation (S.D.). Statistical significance was defined as P < 0.05 unless indicated otherwise. All experiments were repeated a minimum of three times to ensure reproducibility.

## Results

### Identification of Transcriptional Profiles of PBMCs From COVID-19 Patients and Healthy Subjects

The PBMCs from 27 severe COVID-19 patients, 26 mild COVID-19 patients, and 24 healthy subjects were collected. The clinical attributes including demography and blood routine examination are provided in **Table 1** and **Table S1**. Blood counts from patients in the severe group showed lymphopenia (lymphocyte count <1.0* 109/L). Compared to mild patients, severe patients also had significantly higher monocyte and neutrophil proportions (**Table 1**).

**Figure 1:**
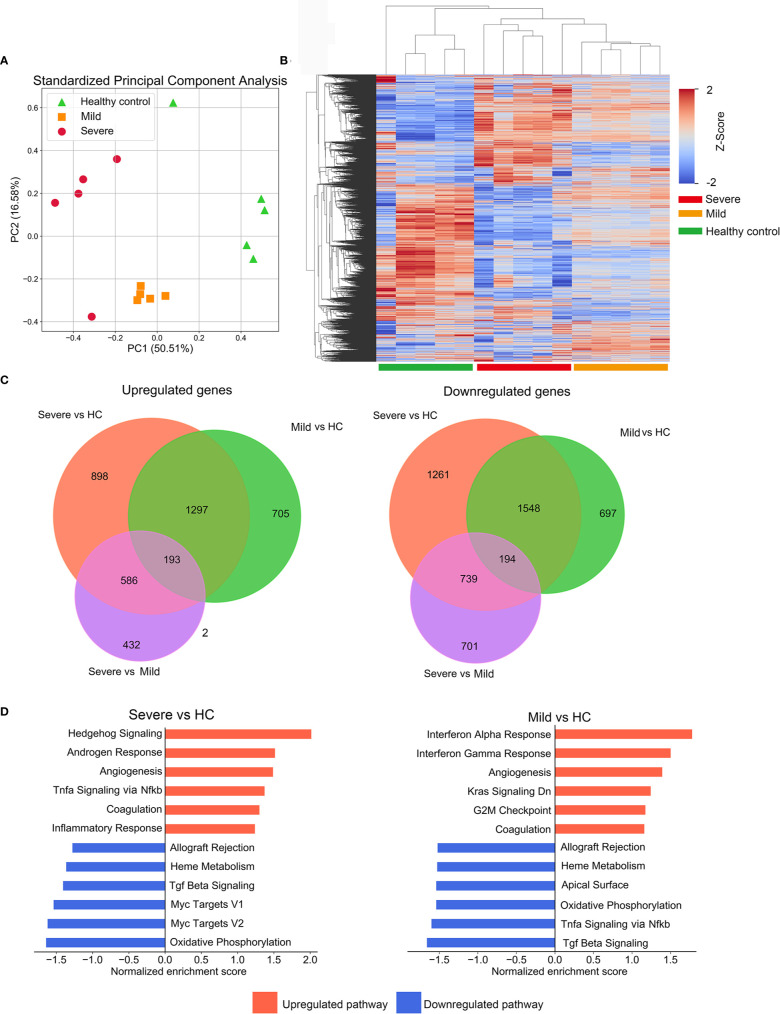
Description of microarray based global gene expression profiles. **(A)** Results of a principal components analysis (PCA) on the expression levels of the global transcriptional genes derived from PBMC samples from all participants. Factor scores are plotted for each participant, with colors differentiating members of the mild, severe patient groups and the healthy control (HC) group. **(B)** Clustered heatmap depicting the expression levels of global genes in the mild and severe patients groups and the HC group. Each row represents a separate gene and each column represents a separate individual. **(C)** Venn diagrams of upregulated or downregulated DEGs in mild, severe and HC groups. The diagrams indicate the numbers of DEGs shared or unique between pairs of groups. **(D)** The enrichment of pathways in severe versus HC and mild versus HC was performed by GSEA. The top enriched pathways are shown (adjusted p-value<0.1).

In order to study the transcriptional profiles associated with SARS-CoV-2 infection, a whole genome microarray analysis was conducted on the PBMCs of 5 severe patients, 5 mild patients, and 5 HCs (**Table S1**). Other participants were included in the validation cohort. About 8,700 DEGs were identified in our microarray analysis (**Table S2**). The results of principal component analysis and cluster analysis of global genes indicated that severe and mild patients groups formed distinctive clusters, which were both separated from the HC group (**Figures 1A, B**). The DEGs between two groups were identified separately (**Figure 1C**, **Table S2**). These results not only revealed the unique transcriptional signatures in patients infected with SARS-CoV-2 compared to HCs, but also indicated the highly distinguished expression pattern between severe and mild infections.

We next employed GSEA with MSigDB hallmark gene sets to characterize their biological pathways. For the severe group, enriched pathways included TNFα signaling and inflammatory response, which implied a robust activation of innate immune and inflammatory signaling. The mild group presented with a significant enrichment in pathways of interferon-α/γ responses, indicating significant elevation of interferons-mediated anti-virus activity. Intriguingly, compared with the HC group, coagulation and angiogenesis were presented in the top upregulated processes in both mild and severe groups, consistent with previous reports that some patients with severe COVID-19 present with coagulopathy and intussusceptive angiogenesis (12, 13) (**Figure 1D**).

### Deregulated T Cell Function and Innate Immune Response in Severe COVID-19 Patients

**Figure 2:**
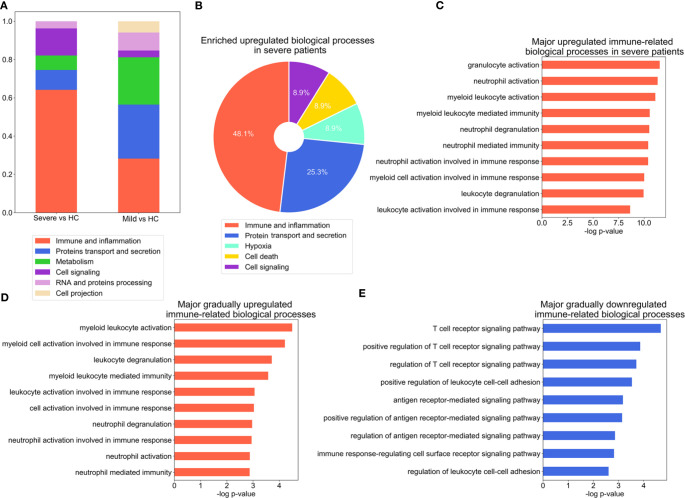
Significant alteration of inflammation- and antiviral immune response-associated biological processes during the COVID-19 course. **(A)** Stacked bar diagram depicting the categories of major biological processes enriched in DEGs, as identified with GO analysis (p-value ≤ 0.001), for severe versus healthy control (HC) and mild versus HC group comparisons. **(B)** Composition of the categories of major biological processes enriched in DEGs in the severe group. The enriched biological process terms were determined using the threshold p-value ≤ 0.01. **(C–E),** Bar charts depicting enrichment of the top immune related-biological processes for the DEGs which are specifically upregulated in the severe group **(C)**, incrementally upregulated across severity groups **(D)**, or incrementally downregulated across severity groups **(E)**. The enriched biological process terms were determined with using threshold p-value ≤ 0.01.

To identify the major biological processes that drive the progression of COVID-19, we performed Gene Ontology (GO) enrichment analysis on DEGs. The major GO annotations for the DEGs were manually classified into five major categories with similar biological processes. Immune and inflammation associated biological processes emerged as most significantly enriched in the severe versus the HC group. In contrast, protein transport and secretion along with immune and inflammation were comparably enriched in the mild versus the HC group (**Figure 2A**, **Figure S1a, S1b**, **Table S3**). To further detail the immune signatures in severe patients, GO enrichment analysis was applied to the DEGs that were specifically presented in the severe group (**Figures 2B, C**, **Figure S1c, S1d** **Table S3**). Indeed, the category of immune and inflammation was most significantly upregulated in the severe group (**Figure 2B**, **Table S3**). Intriguingly, the most enriched GO annotations included in the category of immune and inflammation were the activation of myeloid cells (**Figure 2C**). Whereas, the most significantly downregulated biological processes were associated with ion transport and homeostasis (**Figure S1c**). Additionally, the primary enriched immune-related GO terms were largely related to innate immune response (**Figure S1d**). We also analyzed DEGs upregulated or downregulated in both mild versus HC and severe vs mild group comparisons (**Table S2**). The enriched annotations of genes that were incrementally upregulated from the HC to severe group had similar properties to those of DEGs that were specifically upregulated in the severe group which mainly consisted of myeloid leukocyte function (**Figure 2D** and **Figure S1e**, **Table S3**). Notably, the enriched annotations of genes that were incrementally downregulated from the HC to severe group were mostly related to T cell functions, including T cell receptor signaling and antigen receptor-mediated signaling pathways (**Figure 2E** and **Figure S1f**, **Table S3**). Collectively, these results indicate a substantially impaired immune response in patients infected with SARS-CoV-2.

### Immune Cell Gene Signatures for Profiling the Severe Progression of COVID-19

**Figure 3:**
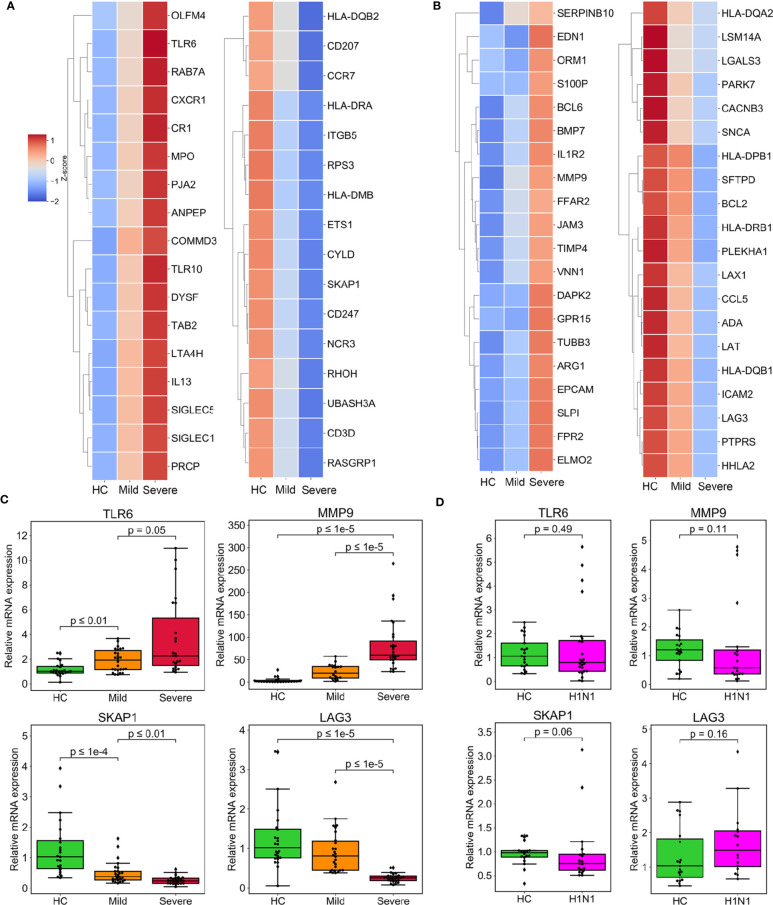
Expression of DEGs specifically enriched in biological processes of immune response associated with severe progression of COVID-19. **(A)** Heatmaps depicting the expression of incrementally upregulated or downregulated DEGs involved in immune and inflammation processes. **(B)** Heatmaps representing the expression of immune- and inflammation-associated DEGs specifically upregulated or downregulated in the severe group. **(C, D)** The results of the validation of relative mRNA expression of TLR6, SKAP1, MMP9, and LAG3 conducted by RT-qPCR in COVID-19 patients **(C)** or H1N1 patients **(D)**, compared with HCs. GAPDH expression was used for normalization. The box plots present the relative normalized intensity scaled by the mean value of the HC group. 50% interquartile ranges are shown in box plots. The medians of each group are denoted as the horizontal lines in the boxes. The excluded outliers are marked as dots. The maximum and minimum values excluding outliers are presented as whiskers. p-values were calculated using the Mann-Whitney U test.

In an effort to identify therapeutic targets and biomarkers which can determine the clinical severity of COVID-19, we looked in detail at the DEGs associated with immune function which had been found to either be regulated among the groups incrementally (**Table S4**, **Figure 3A**) or be specifically regulated in the severe group (**Table S4**, **Figure 3B**). Interleukin 13 (IL-13), which has been previously found to be reported tightly closely associated with increased airway hyperreactivity and prolonged pulmonary responses was the most significantly affected among the incrementally upregulated DEGs (14). In particular we note the involvement of myeloperoxidase (MPO), a component protein of azurophilic granules released by neutrophils which is a crucial player in inflammatory and immune response (15). Other relevant genes including C-X-C motif chemokine receptor 1 (CXCR1), sialic acid binding Ig like lectin 5/14 (SIGLEC5/14) and leukotriene A4 hydrolase (LTA4H) are part of a subset of genes responsible for facilitating inflammatory response by the recruitment and chemotaxis of neutrophils (16–18). Interestingly, toll-like receptor 6/10 (TLR6/10), which have been well implicated in immune response to bacteria and fungi were also upregulated, suggesting they may play a role in responding to SARS-CoV-2 infection. The DEGs that were specifically upregulated in the severe group included matrix metallopeptidase 9/25 (MMP9/25), which are secreted by neutrophils and macrophages to promote their transendothelial migration to inflammatory sites (19, 20). Endothelin 1 (EDN1), which encodes the cytokine that mediates respiratory inflammation, was also induced (21, 22). Notably, recent study underlined EDN1 as a unique biomarker of SARS-CoV-2 infection when compared to other respiratory viruses such as seasonal influenza A virus (IAV) and human respiratory syncytial virus (RSV) (23). In addition, inflammatory antagonist interleukin 1 Receptor Type 2 (IL1R2) and secretory leukocyte peptidase inhibitor (SLPI) were significantly upregulated in severe patients, suggesting the initiation of a negative feedback loop due to the excessive inflammatory response (**Figure S2a**). The DEGs incrementally downregulated across severity groups included Src kinase associated phosphoprotein 1 (SKAP1) and lymphocyte activation gene-3 (LAG-3) that both play critical roles in T cell activation (24). Additionally, DEGs downregulated incrementally or specifically in the severe group included HLA class II paralogs such as HLA-DQB1/2, HLA-DMB, HLA-DRA, and HLA-DPB1, suggesting that increasing disease severity impairs the function of presenting antigens to T cells in antigen-presenting cells (**Table S4**, **Figure S2b**).

### The Specifically Regulated Immune Genes Associated With Severe COVID-19

We validated our interpretation of the selected genes above by conducting RT-qPCR of PBMCs in the validation cohort. The four genes, TLR6, SKAP1, MMP9, and LAG3 all showed a consistency with microarray data (**Figure 3C**). We also constructed PPI networks based on the STRING database. LAG3 and SKAP1 showed a strong association with DEGs that were enriched in T cell activation (**Figures S3a, S3b**). Additionally, TLR6 was identified as the hub protein of the DEGs associated with cytokine production (**Figure S3c**). Finally, MMP9 was related to genes that regulate granulocyte activation (**Figure S3d**).

To ascertain whether the regulation of these genes is unique to SARS-CoV2 infection, we determined their expression pattern in the PBMCs of patients infected with H1N1 virus. Of interest, compared with an independent cohort of healthy subjects, all four of the test genes were not up-regulated or down-regulated by H1N1 infection (**Figure 3D**).

**Figure 4:**
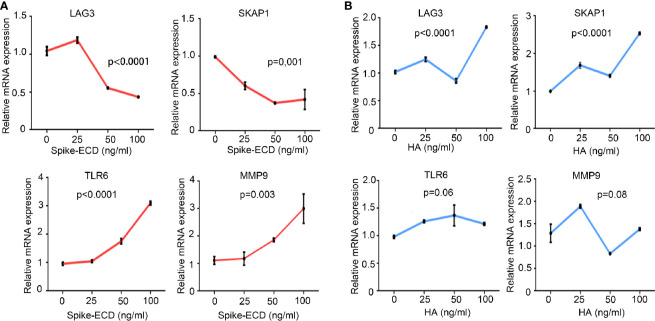
The regulation of LAG3, SKAP1, TLR6, and MMP9 by Spike-ECD, but not HA e*x vivo*. **(A, B)** PBMCs from healthy donors were combined with Spike-ECD **(A)** or HA **(B)** at a concentration of 0, 25, 50, or 100 ng/ml for 12 h. Cells were then harvested and the expression of indicated genes was measured by RT-qPCR. GAPDH expression was used for normalization. n = 3 independent experiments. Data are presented as mean ± S.D. p-values were calculated using a one-way ANOVA for multiple comparison.

We also stimulated PBMCs from healthy subjects with either an extra-cellular domain truncation of SARS-CoV-2 Spike protein (Spike-ECD) or Hemagglutinin (HA) protein from Influenza A H1N1 (A/California/04/2009), both of which belong to the major immunity-eliciting antigens and mediate the invasion of virus to host cells. Our results demonstrated that Spike-ECD upregulated the expression of TLR6 and MMP9 and downregulated the expression of LAG3 and SKAP1 *ex vivo*, which were in agreement with our microarray results (**Figure 4A**). In contrast, HA from H1N1 virus had no significant effect on these genes (**Figure 4B**).

In order to explore which type of immune cells in PBMCs responds to the stimulation of Spike, resulting in the alteration of the expression of specific genes, we stimulated THP-1 cells, a human monocytic cell line with Spike-ECD, and then co-cultured them with HuT 78 cells, which have the properties of a mature human T cell with helper/inducer activity. The results suggested that the upregulation of MMP9 and TLR6 by Spike were mainly occured in THP-1 cells, which consisted with our GO analysis that myeloid cells, which contain monocytic cells were activated in COVID-19 patients. SKAP1 and LAG3 were mostly expressed in HuT 78 cells. The expression of LAG3 was slightly downregulated in both HuT 78 and THP-1 cells upon Spike stimulation. While, the expression of SKAP1 was not regulated by Spike in either of these two cell lines (**Figure S4a**).

### Inadequate Response of the Type I IFNs Signaling in Severe COVID-19 Patients

**Figure 5:**
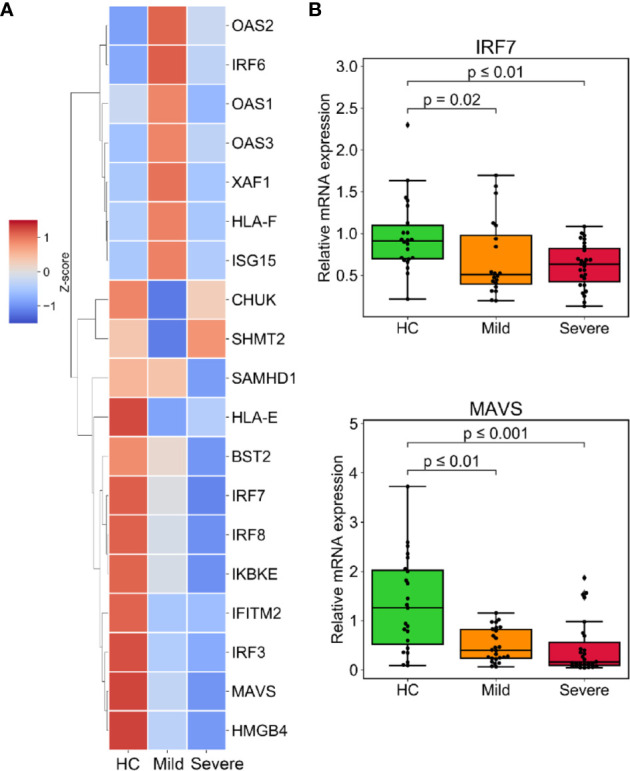
Muted response of the type I interferons (IFNs) signaling associated with severe progression of COVID-19. **(A)** The expression level of downregulated DEGs involved in IFN secretion (GO:1902741) or responding to type I IFNs (GO:0034340). **(B)** The relative mRNA expression of indicated genes was validated by RT-qPCR in the validated cohort. GAPDH expression was used for normalization. p-values were calculated using the Mann-Whitney U test.

Type I IFNs signaling plays a crucial role not only in combating microorganisms, but also in limiting excessive neutrophil and monocyte activation and counteracting the hyperinflammatory response (25, 26). Given this, we examined DEGs that promote IFNs secretion and response to type I IFNs (GO: 1902741 and GO: 0034340). Surprisingly, the progression of the disease was followed by the downregulation of IFN-stimulated genes (ISGs) and genes that crucially regulate type I IFNs, including IRF3/7/8 and MAVS (**Figure 5A** and **Figure S5a**). The expression of some ISGs like OAS1/2/3 and the upstream regulator of the IFNs pathway IRF6, were unresponsive in patients with severe infection (**Figure 5A** and **Figure S5b**). We also confirmed the downregulation of IRF7 and MAVS in the patients group from the validation cohort (**Figure 5B**).

### The Alteration of Immune Cells Composition in Consistent With Gene Signatures in COVID-19 Patients

**Figure 6:**
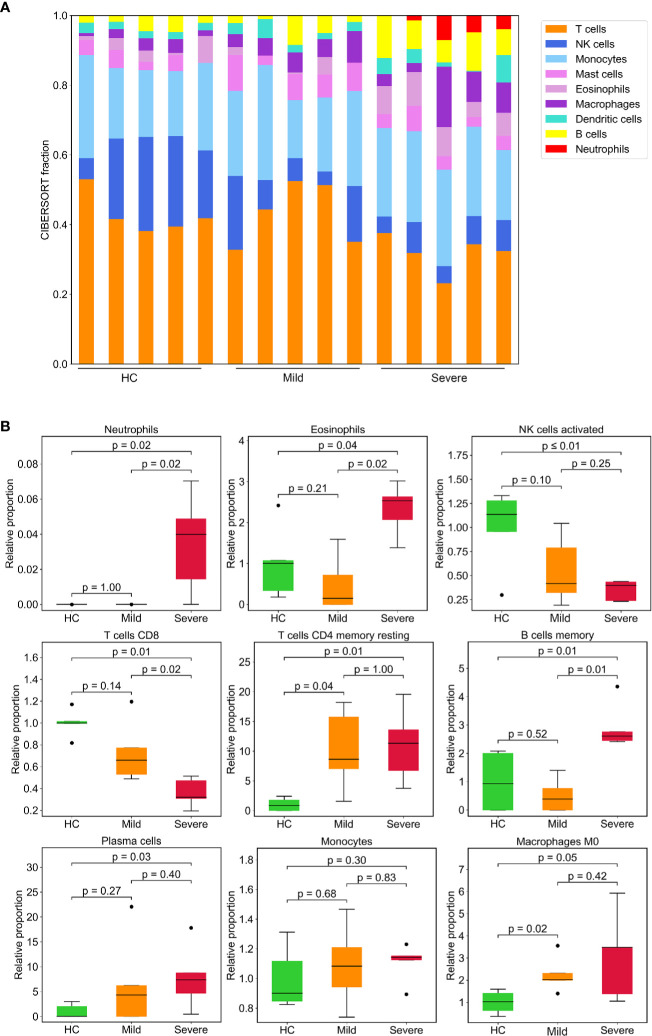
Leukocyte composition evaluated by CIBERSORT based on gene expression. **(A)** Stacked bar diagram showing the proportions of nine different types of immune cells in patients and HCs. Each bar represents an individual. **(B)** Immune cell fractions were determined for each group. The relative normalized intensity was scaled by the mean value of the HC group. p-values were calculated using the Mann-Whitney U test.

Given that the altered proportion of lymphocytes may contribute substantively to the unique immune gene expression signatures of PBMCs from patients with SARS-CoV-2. Thus, we estimated the composition of different immune cell types using CIBERSORT, a versatile computational method for quantifying cell fractions from gene expression profiles (27). We found a marked increase in the proportion of neutrophils in the severe patient’s group, consistent with results of the blood routine and our analysis of gene expression which demonstrated that myeloid cells were robustly activated in COVID-19 patients. This analysis also revealed that cytotoxic CD8^+^ T cells decreased as the disease severity increased. Additionally, there was a smaller proportion of activated NK cells in severe patients compared to the HC group. Consequently, IFN-γ, which is mainly produced by NK cells and T cells, was downregulated in patients (**Table S2**). The proportion of B cells, especially memory B cells, was higher in the severe group, CD4+ memory T cells were elevated in patients (**Figures 6A, B**).

## Discussion

In our study, we used GO analysis to profile the peripheral immune response in patients based on their whole genome transcriptome. We found that DEGs regulated incrementally by disease severity or uniquely regulated in patients with severe infection were enriched on in biological processes related to myeloid cell activation and deficits in T cell function; these findings concur with clinical features of severe infection, including neutrophil filtration, lymphopenia and T cell exhaustion. It is noteworthy that although most SARS-CoV-2 infection occurs in the lower respiratory tract, there is suggestive evidence that SARS-CoV-2 also presents in PBMCs, and may contribute to lymphocytopenia (28, 29). Thus, examining the transcriptomic profiles of PBMCs will help reveal the unique expression of the host response to SARS-CoV-2 in COVID-19 patients.

In our study, we identified the four most significantly regulated immune-related genes, TLR6, SKAP1, MMP9, and LAG3 and validated them in a larger cohort. Using PPI analysis, LAG3 and SKAP1 were found to have a strong association with DEGs that were enriched in processes involved in T cell activation, and TLR6 and MMP9 were identified as critical proteins for the function of myeloid cells. In keeping with these findings, we also verified *ex vivo* that the expression pattern of these genes was uniquely presented in SARS-CoV-2 infection and largely mediated by Spike protein. These results implied the regulation of these 4 genes could at least partly reflect the unique regulation signatures underpinning COVID-19 progression. Given that whole blood or PBMCs are more easily obtained from patients compared to other infected tissues, these target genes could be examined as potential diagnostic markers for disease severity, though these regulated genes should be validated in a larger cohort.

Neutrophils act as the first line of immune defense against pathogens. However, excessive inflammatory response induced by immune cells such as neutrophils and monocytes leads to potentially fatal hypercytokinemia, which has been implicated in SARS infection (30). Notably, the presence of the neutrophil-to-lymphocyte ratio was identified as an independent risk factor for severe illness (3, 31). In addition, neutrophil infiltration in pulmonary capillaries, acute capillaritis with fibrin deposition, extravasation of neutrophils into the alveolar space, and neutrophilic mucositis were observed in different independent reports on the pathological findings from autopsied COVID-19 patients (32, 33). We identified a plethora of genes upregulated in patients which are responsible for the recruitment of neutrophils and mediate the proinflammatory function of neutrophils. This finding suggests that neutrophils may be the main contributor to disease progression.

Our analysis suggests a stark muting or downregulation of the type I IFNs response in patients, especially in severe patients. This finding is consistent with recent reports that the response of IFNs is reduced in COVID-19 (23, 34, 35). Viruses have evolved the capacity to suppress the host’s IFNs signaling. SARS-CoV has been reported to block the induction of IFNs (36, 37), further studies are needed to elucidate the mechanisms by which SARS-CoV-2 disrupts IFN-mediated anti-virus activity. Indeed, type I IFNs have been evaluated in clinical trials to treat MERS-CoV and SARS-CoV (38, 39), and IFN treatment has been adopted as a potentially effective antiviral therapy against SARS-CoV-2 infection, although the treatment outcomes are still uncertain (40, 41). Our study contributes to the growing evidence of the therapeutic potential of type I IFNs for treating COVID-19. In sum, our findings not only improve our understanding of the pathogenesis of SARS-CoV-2, but may also provide potential therapeutic targets for treatment, especially for patients in severe or critical stages which have thus far been the most difficult to manage.

## Data Availability Statement

The datasets presented in this study can be found in online repositories. The names of the repository/repositories and accession number(s) can be found below: Gene Expression Omnibus (GEO) and GSE164805.

## Ethics Statement

The studies involving human participants were reviewed and approved by The Clinical Research Ethics Committee of The First Affiliated Hospital, School of Medicine, Zhejiang University. The patients/participants provided their written informed consent to participate in this study.

## Author Contributions

QZ and HD conceived and designed the study. QZ, YM, XZ, and WC performed the experiments and statistical and bioinformatic analysis. QZ, YM, and KW collected and characterized samples. QZ and HD wrote and edited the manuscript. JS, YQ, LL, and HD supervised the study. All authors contributed to the article and approved the submitted version.

## Funding

This work was supported by the major national S&T projects for infectious diseases (2018ZX10301401), the Key Research & Development Plan of Zhejiang Province (2019C04005), and the National Key Research and Development Program of China (2018YFC2000500).

## Conflict of Interest

The authors declare that the research was conducted in the absence of any commercial or financial relationships that could be construed as a potential conflict of interest.
