# Supplementary material for: Inflammation and Antiviral Immune Response Associated With Severe Progression of COVID-19
Source: Front Immunol. 2021 Feb 18;12:631226. doi: 10.3389/fimmu.2021.631226 (PMC7930228; doi:10.3389/fimmu.2021.631226)
Supplement: Supplementary file 1 [file DataSheet_1.zip › Supplementary_ Materials/Supplementary_Figures and Figure Legends.docx]

Supplementary Material

**This supplementary figures and figure legends contains figure S1-S5 and figure legends S1-S5.**

**Figure S1**


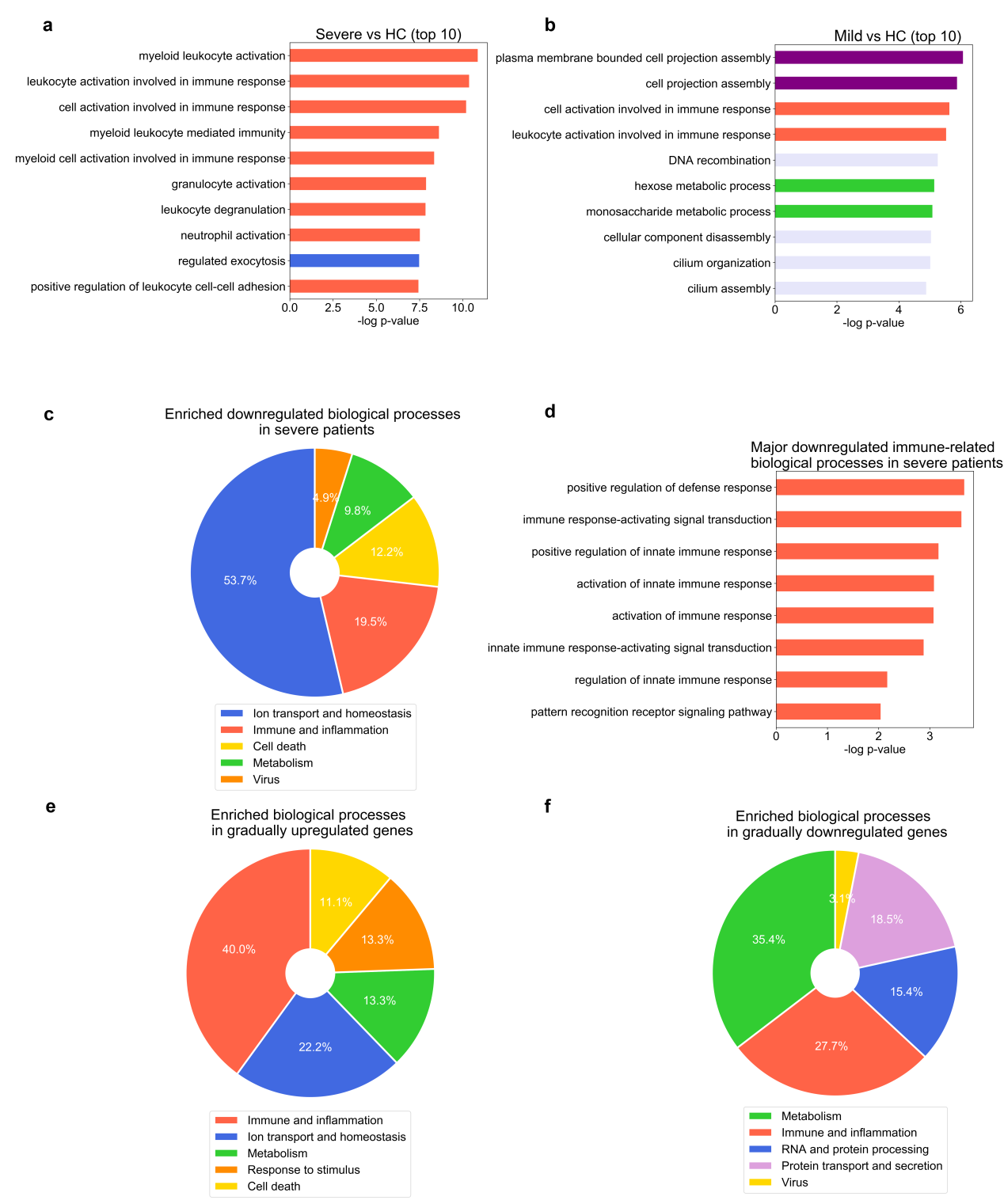


**Figure S2**


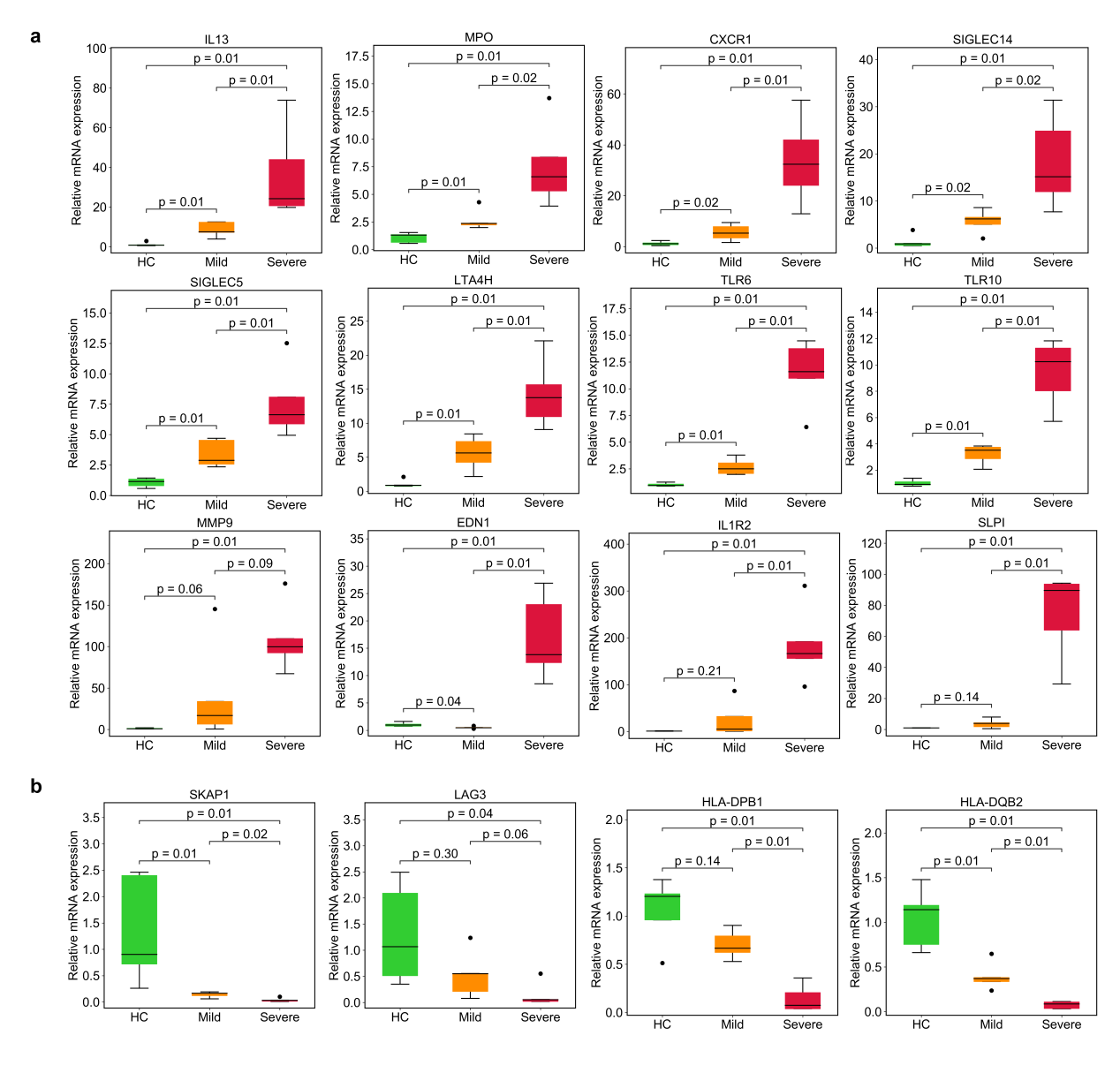


**Figure S3**


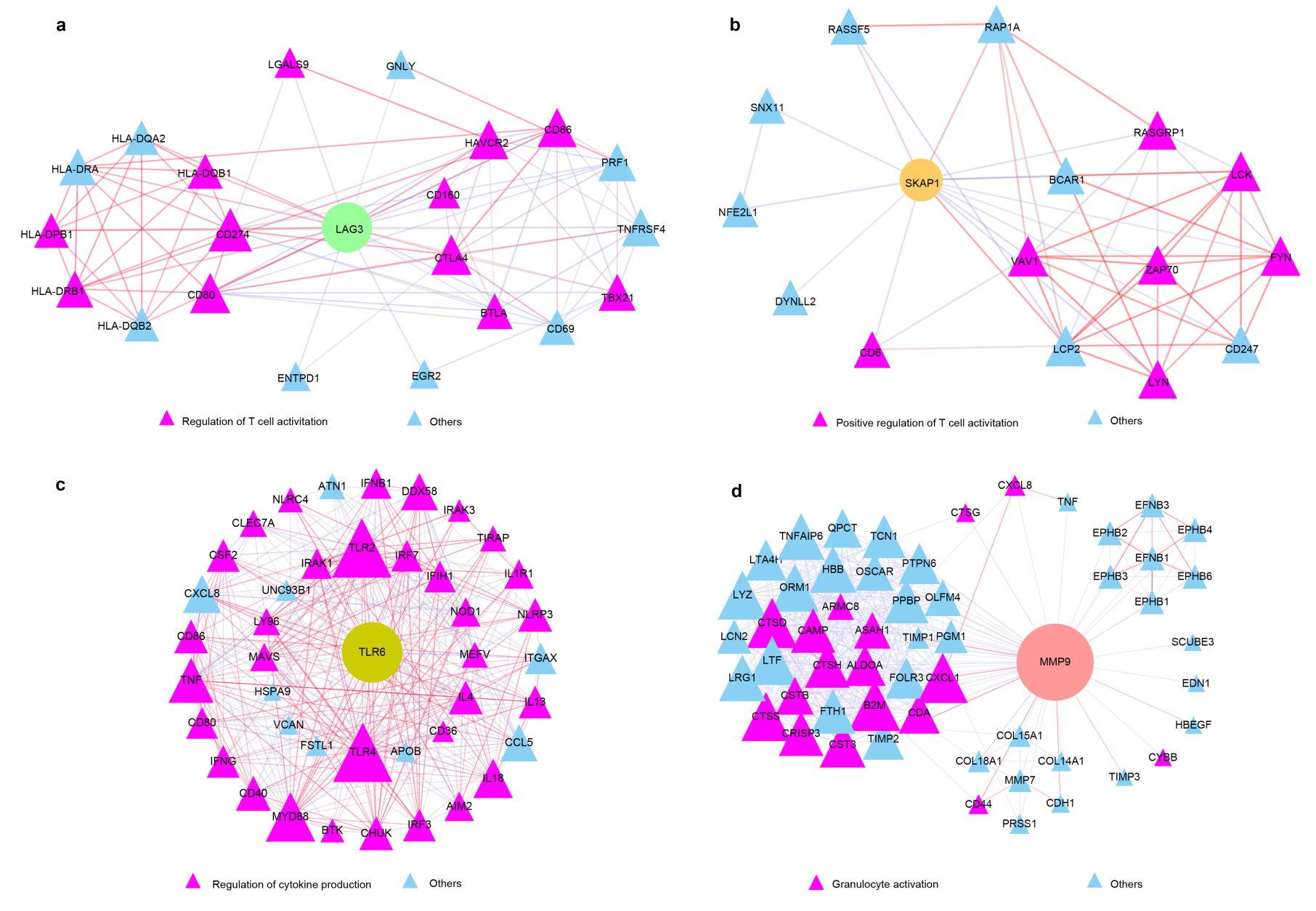


**Figure S4**


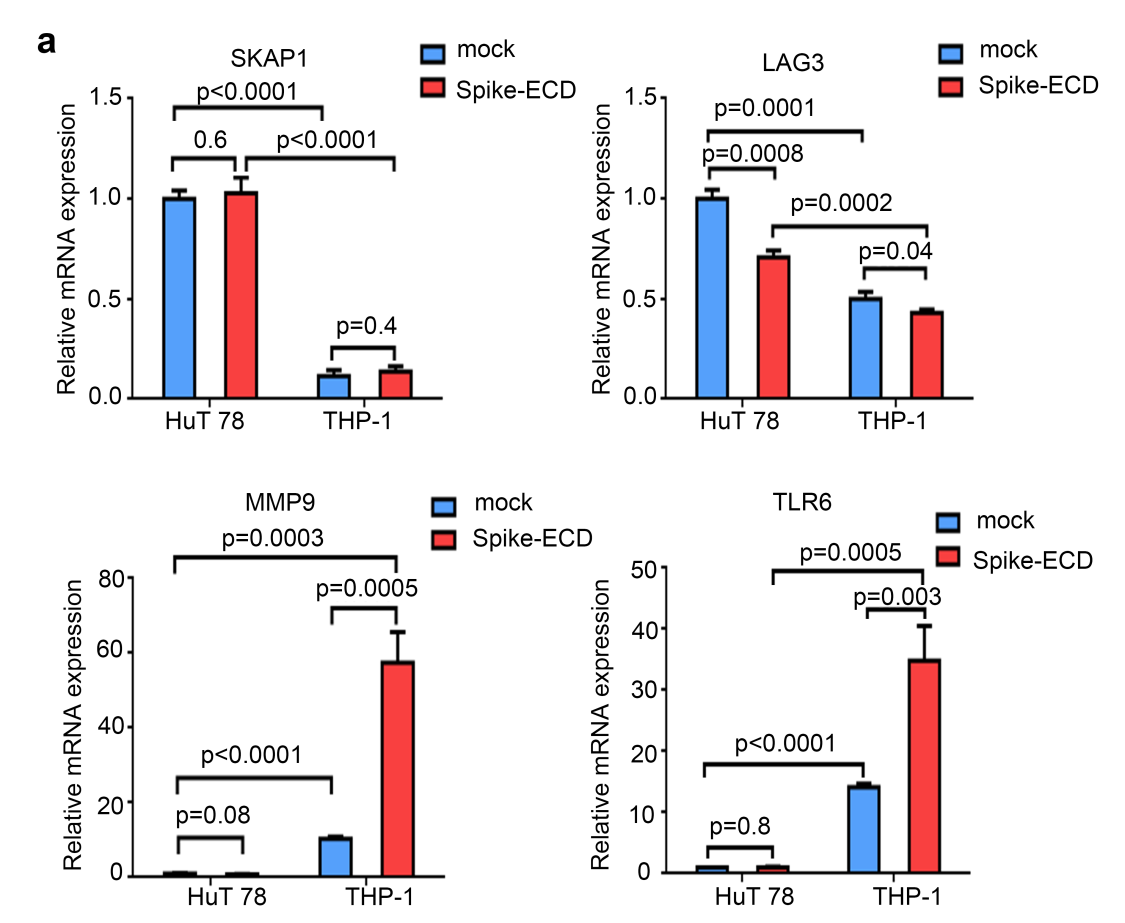


**Figure S5**


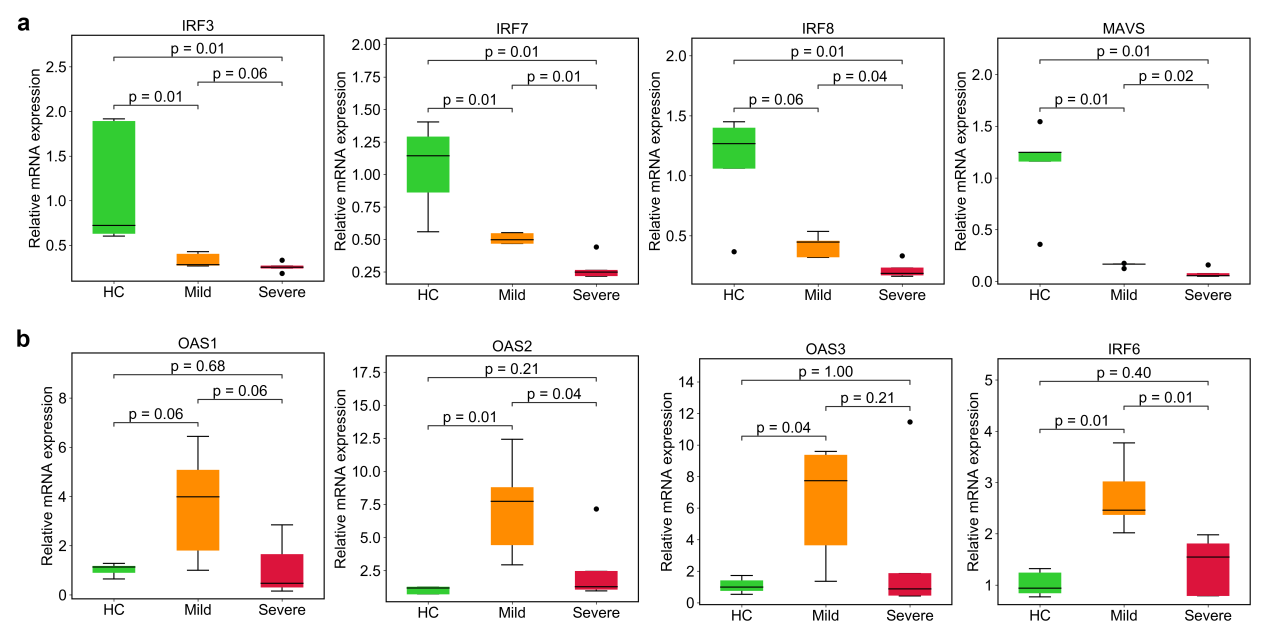


**Supplementary figure legends**

**Figure S1. Biological processes enriched in DEGs of COVID-19 patients compared with HCs.** (a, b) Bar charts depicting the top 10 biological processes enriched in DEGs for the severe versus HC groups (a), and mild versus HC groups (b) with p-value ≤0.001. (c) Pie chart indicating the proportion of the major categories of downregulated biological processes enriched in the severe group. (d) Immune-related biological processes terms were enriched in the downregulated DEGs of the severe group. (e, f) The proportion of the major categories of biological processes enriched in incrementally upregulated (e) and downregulated (f) DEGs are shown. The enriched biological process terms were determined with the threshold p-value ≤0.01.

**Figure S2. Relative expression of DEGs in the category of immune and inflammation, which are associated with the progression of COVID-19.** (a, b) Relative expression levels of the indicated genes, either upregulated (a), or downregulated (b) in the patients groups, are shown as box plot diagrams. The boxes represent the relative normalized intensity scaled by the mean value of the HC group. p-values were calculated using the Mann-Whitney U test.

**Figure S3. Description of biological significance of selected DEGs using PPI network analysis.** (a, b, c, d) PPI networks of LAG3 (a), SKAP1 (b), TLR6 (c) and MMP9 (d) in the DEGs of patients. Each node is representative of a gene. The node size represents the connectivity degree. The colors of the edges indicate the scores: purple = 901-949, grey = 950, red = 951-999. The nodes representing genes included in the most significantly enriched GO term are fuchsia, with the other nodes colored blue.

**Figure S4. The regulation of LAG3, SKAP1, TLR6 and MMP9 by Spike-ECD in HuT 78 and THP-1.** (a) THP-1 cells were stimulated with Spike-ECD for 24 hours, and then co-cultured with HuT 78 for another 24 hours. Cells were then harvested and the expression of indicated genes was measured by RT-qPCR. GAPDH expression was used for normalization. n = 3 independent experiments. Data are presented as mean ± S.D. p-values were calculated using an unpaired t-test.

**Figure S5. Relative expression of DEGs associated with type I IFNs signaling.** (a, b) Relative expression levels of the indicated genes, either dowregulated in patients groups (a), or specially muted in severe group (b), were calculated by normalizing the intensity of each group which was scaled by the mean value of the HC group. p-values were calculated using the Mann-Whitney U test.
